# Tempering Temperament: Exploring the Influence of Maternal Mind‐Mindedness on Infant Temperament in Shaping Socioemotional Wellbeing

**DOI:** 10.1111/infa.70032

**Published:** 2025-07-23

**Authors:** Allira Doyle, Emma E. Walter, Samudra Radhakrishnan, Frances L. Doyle

**Affiliations:** ^1^ School of Psychology MARCS Institute for Brain, Behaviour and Development Western Sydney University Sydney Australia; ^2^ School of Psychological Sciences Faculty of Medicine Health and Human Sciences Macquarie University Sydney Australia

**Keywords:** differential susceptibility, infant temperament, mind‐mindedness, negative affect, sensitive parenting, socioemotional wellbeing

## Abstract

Although several studies independently explore temperament and parenting, research on connections between temperament and mind‐mindedness are largely absent. This study examined relationships between maternal mind‐mindedness and infant temperament on infant socioemotional wellbeing. Participants comprised culturally diverse mother‐infant dyads (*n* = 63; 52.38% girls). Infants, aged 4‐ (*n* = 32) and 8‐months‐old (*n* = 31), and their mothers completed a free‐play task. While direct relationships between mind‐mindedness and wellbeing were not supported, an indirect interactional relationship between mind‐mindedness and temperament on wellbeing was supported. Mind‐mindedness moderated the relationship between negative affect and socioemotional development whereby infants with higher negative affect who received higher appropriate comments had better socioemotional wellbeing than their lower negative affect counterparts. This highlights that, for higher negative affect infants, appropriate comments are particularly influential in enhancing wellbeing. Effortful control predicted wellbeing, suggesting that specific temperament traits experience optimal socioemotional development independently of mind‐minded parenting. These findings broaden research knowledge regarding the differential susceptibility hypothesis. Overall, this study has shown how wellbeing can be affected by the temperamental dispositions that infants bring into the world as well as the parenting experiences that they encounter.

## 1 Introduction

Socioemotional development is essential for wellbeing and has many complex extrinsic and intrinsic influences. Sensitive caregiving, perhaps the most crucial external factor upon development (Collins et al. 2000; Sameroff 2010), manifests as warm and responsive nurturing. Hence, it fosters effective regulation and optimal attachment, which are core precursors to socioemotional wellness (Hwa‐Froelich 2022). Mentalization, the process by which parents understand and interpret their child's mental and emotional states, is considered salient for parental sensitivity (Zeegers et al. 2017). Within this context, mind‐mindedness—a parent's ability to recognize and comment on their child's internal states (Meins 1997; Meins et al. 1998, 2001)—serves an important function through adept attunement to unique perspectives and inner experiences (Meins 1999). Mind‐mindedness actively promotes attachment, theory of mind, and self‐regulation (McMahon and Bernier 2017). In contrast, temperament serves as an internal determinant of socioemotional outcomes; it concerns innate characteristics of emotional intensity, activity level, and regulation abilities (Sanson et al. 2004). Distinct temperamental profiles have been associated with divergent social and emotional trajectories (Abulizi et al. 2017; Kostyrka‐Allchorne et al. 2020). While inherited traits establish brain capacity, early experiences affect the connection and expression of these foundations, highlighting infancy as critically formative for supporting this biological and social interaction (Hwa‐Froelich 2022). Both mind‐mindedness and temperament significantly impact socioemotional development, yet the research exploring the intricate interplay between these factors remains relatively scant. Consequently, whether mind‐mindedness may moderate the relationship between infant temperament and socioemotional development is unclear.

### 1.1 Socioemotional Development

Socioemotional development is multifaceted and includes progressive abilities to experience, process, and express the emotional spectrum; foster meaningful relationships; and learn through the surrounding environment (Pontoppidan et al. 2017). Zilanawala et al. (2019) broadly defines poor socioemotional outcomes as internalizing (i.e., inhibited behavior characterized by withdrawal and isolation) and externalizing issues (i.e., disinhibited behavior characterized by defiance and hostility). Both represent forms of emotional dysregulation, albeit with varying degrees of observability (Cooke et al. 2022). It then follows that regulatory abilities are crucial for optimal socioemotional development.

Regulation entails emotional and cognitive integration to coordinate goal‐directed pursuits (Mischel and Ayduk 2002). Difficulties in childhood regulation are often associated with future socioemotional, behavioral, and academic issues (Thomas et al. 2019). Key regulatory functions begin in infancy (Grolnick et al. 1996), with parenting shown to influence children's emotional arousal (Fabes et al. 2001). Parental supportiveness (i.e., expressing comfort and naming emotions) can decrease (Lougheed et al. 2015), and unsupportive parenting (i.e., becoming angry or punitive) can increase (Frick and Morris 2004) children's negative emotional arousal. Taken together, this suggests that parent‐child relationships represent the nexus of child‐stress response regulation (Bernier et al. 2010).

Caregivers function as the secure base from which infants explore, providing reassurance that essential physical and psychological needs will be satisfied (Ainsworth et al. 1978). Parental responsiveness to cues and accuracy in responding create neural expectations for future social interactions, providing an optimal template for later outcomes (Hwa‐Froelich 2022; Luyten et al. 2020). Ergo sensitivity to infant states is paramount. Sensitivity requires parents' awareness of their child's perspective by swiftly and appropriately identifying and interpreting various cues (Ainsworth et al. 1974). Mentalization, which involves recognizing the mental agency of others to connect observable inner states with observable outer behaviors, is considered a component of sensitivity as it assists parents' understanding of and responsiveness to the emotional and mental states of their children (Senehi et al. 2018; Yatziv et al. 2018).

### 1.2 Mind‐Mindedness

Considered a mentalization skillset, mind‐mindedness involves parents recognizing the internal states of their children and manifests as spontaneous comments during dyadic interactions (Meins 1997; Meins et al. 1998, 2001). It operates through verbal tools to extract mental events (Meins et al. 2013). For example, mind‐mindedness comments could include “You want this toy” and “You're bored of that toy.” Accordingly, mind‐mindedness is multidimensional and measured as appropriate or non‐attuned depending on whether interpretations are congruous or incongruous to infant behavior, respectively (Meins and Fernyhough 2015). Dimensions are uncorrelated and likely tap into different elements of mind‐mindedness (Yatziv et al. 2018).

Research broadly supports mind‐mindedness as independently influencing advantageous developmental outcomes (Zeegers et al. 2017); such as brain connectivity (Dégeilh et al. 2018), language acquisition (McMahon and Bernier 2017), school readiness (Bernier et al. 2017), academic performance (Meins et al. 2019), and interpersonal skills (Colonnesi et al. 2019). Strong converging evidence has also been established for associations between mind‐mindedness and secure attachment (Aldrich et al. 2021; Arnott and Meins 2007; Laranjo et al. 2008; Laranjo et al. 2010; Lundy 2003; McMahon and Bernier 2017; Meins et al. 2012). Conversely, poorer mind‐mindedness predicts insecure attachment styles (Bigelow et al. 2018; Meins et al. 2018; Shai and Meins 2018). Regulatory skills have likewise been consistently linked to mind‐mindedness (Bernier et al. 2010), with fewer externalizing issues (Dollberg et al. 2021) and fewer internalizing issues (Hobby et al. 2022) associated with mind‐minded parenting. It follows that inadequate mind‐related comments are related to internalizing and externalizing problems (Meins et al. 2013). Research has not, however, considered variations in outcomes regarding individual infant differences, namely temperament.

### 1.3 Temperament

Temperament is the inherent individual variations influencing responses to stimuli and regulation of self (De Pauw and Mervielde 2010). Rothbart (1981) proposed a theoretical model whereby latent psychobiological mechanisms determine temperamental differences resulting in distinctive affective, activation, and attentional patterns (Gartstein and Rothbart 2003; Rothbart et al. 1995). Such patterns significantly impact personality qualities and social behaviors throughout life (Rothbart et al. 1995), through automatic neural reactivity and controlled regulatory adjustment (Posner and Rothbart 2009). Given its comprehensive psychobiological approach, this model was selected for the current study.

Rothbart and Bates (2006) organize temperament into three broad domains including negative affect, surgency, and effortful control. Negative affect comprises the propensity toward experiencing negative emotions (i.e. irritability, sadness, limited soothability, discomfort with novelty). Surgency relates to social disposition and motor activity (i.e., activity level, sociability, unreservedness, impulsivity). Effortful control is shown through attentional and inhibitory shifting capacities (Rothbart et al. 2003).

Ample evidence connects temperament and socioemotional developmental outcomes (Abulizi et al. 2017; Sanson et al. 2004; Kostyrka‐Allchorne et al. 2020). High negative affect transdiagnostically increases risks for the development of later psychopathology and poor developmental outcomes. It predicts both internalizing (Gartstein et al. 2012; Abulizi et al. 2017) and externalizing problems (Sirois et al. 2019; Yavuz‐Müren et al. 2022Yavuz‐Müren et al. 2022). Low surgency relates to later social impairment (Dollar and Stifter 2012) and internalizing symptoms (Gartstein et al. 2012); whereas high surgency relates to externalizing problems (Abulizi et al. 2017) and attention‐deficit/hyperactivity disorder (Kostyrka‐Allchorne et al. 2020). Low effortful control increases risks for later psychopathology, autism spectrum disorder, attention‐deficit/hyperactivity disorder diagnoses (Kostyrka‐Allchorne et al. 2020), and externalizing problems (Eisenberg et al. 2010); while high effortful control increases social competence (Laible et al. 2014) and internalizing problems (Yavuz‐Müren et al. 2022Yavuz‐Müren et al. 2022).

Temperamental expression is impacted by individual experiences (Rothbart et al. 2011), epigenetic influences (Gartstein and Skinner 2018), social environments (Braungart‐Rieker et al. 2010), and parenting practices (Ryan and Ollendick 2018). Accordingly, the diathesis‐stress model holds that psychopathology develops in those with increased genetic sensitivities to environmental influences (Zuckerman 1999). Thus, misaligned parenting practices may more detrimentally impact infants with temperamental vulnerabilities (Ryan and Ollendick 2018). Moreover, differential susceptibility theory expands upon this premise by postulating that while certain temperamental profiles are more negatively impacted by stressors, they are also more positively impacted by supports (Belsky et al. 2007; Belsky 2005). Under this theory, gene‐environment interactions bias toward advantage or disadvantage (Belsky et al. 2007; Bradley and Corwyn 2002; Dunsmore et al. 2016; Slagt et al. 2016; Stoltz et al. 2017). Certain temperaments may have heightened susceptibility to the protective or detrimental effects of nurturing versus aversive parenting (Davies et al. 2021).

### 1.4 Parenting and Temperament

Parenting powerfully influences temperament‐development outcomes (Lengua et al. 2019). High negative affect infants surpass less negative affect infants on self‐regulatory measures when securely attached (Kim and Kochanska 2012). High negative affect infants experience greater socioemotional outcomes in childhood when sensitively parented than their counterparts who are not sensitively parented (Zhang et al. 2022). Low surgency children exposed to positive parenting show better emotion regulation (Yagmurlu and Altan 2010), while high surgency children exposed to negative parenting encounter more externalizing issues (Brown et al. 2022; del Puerto‐Golzarri et al. 2022; Ruiz‐Ortiz et al. 2023). Low effortful control children receiving low sensitive parenting experience greater internalizing and externalizing problems (Slagt et al. 2016), while high effortful control children receiving low authoritative parenting experience greater aggression (del Puerto‐Golzarri et al. 2022). Following differential susceptibility theory, infant socioemotional development may be differentially impacted by mind‐minded parenting, depending upon temperamental factors.

### 1.5 Mind‐Mindedness and Temperament

No studies have specifically investigated the moderating role of mind‐mindedness on the relationship between temperamental factors and socioemotional outcomes. Research regarding interactions between mentalization and temperament is mixed. Some studies indicate temperament hinders mentalization efforts; Demers et al. (2010) found that difficult temperament, defined as a negative parental perception of infant disposition, at 12 months has been linked with reduced mind‐mindedness at 18 months. Conversely, other studies find that mentalization is unaffected by temperament; Meins et al. (2011) found no relationship between temperament and mind‐mindedness between 3 and 7 months. Despite these findings, recent studies provide preliminary theoretical and empirical support for links between temperament and mind‐mindedness.

There have been some examinations of maternal mind‐mindedness and infant expression of negative affect. With respect to mind‐mindedness, appropriate comments are congruent with infant behaviors, whereas non‐attuned comments are incongruent with infant behaviors (Meins and Fernyhough 2015). McMahon and Newey (2018) found that higher non‐attuned comments were related to more intense infant reactions (i.e., excessive crying or absent affect). Mothers of infants who displayed more distress earlier in the interaction uttered greater appropriate mind‐related comments in subsequent interactions, whereas mothers of infants who displayed less distress earlier in the interaction uttered fewer appropriate mind‐related comments in subsequent interactions. Researchers speculated that signals of distress might have incentivized mothers to remain sensitively engaged and accurately interpret cues. Relatedly, Planalp et al. (2019) found that parents practised more mind‐mindedness (i.e., higher frequency of mind‐related comments, both appropriate and non‐attuned) and made more non‐attuned comments when infants displayed greater negative affect. Researchers suggested that parental attunement to infant cues might thus buffer the adverse impacts of negative affect on attachment style. While both studies focused on observable rather than temperamental negative affect, results highlight that infant behaviors are complexly linked with parental behaviors. Thus, parental mind‐mindedness appears to influence, and be influenced by, infant affect.

Nikolić et al. (2022) examined whether mind‐mindedness affects children's regulation. Mind‐related comments were coded during free play sessions at 12‐months‐old and 30‐months‐old, with temperamental regulation measured at 4.5 years. Results showed that paternal appropriate mind‐related comments were related to higher regulation while parental non‐attuned mind‐related comments were related to lower regulation––implying an interrelationship between mind‐mindedness and temperament. Ergo differences in parental factors (i.e., mind‐mindedness) and differences in infant factors (i.e., regulation) jointly shaped developmental outcomes.

Zilanawala et al. (2019) outline that mind‐mindedness divergently impacts individuals with tendencies toward internalizing or externalizing behaviors. Findings revealed that internalizing‐prone infants benefited more from appropriate mind‐related comments than externalizing‐prone infants, who were more likely to engage in challenging behaviors. Whilst these results do not allow for direct comparisons with temperament, it is suggestive that different infant dispositions may interact with mind‐mindedness in different ways.

### 1.6 The Current Study

As infant characteristics influence parenting practices and parenting practices shape later developmental outcomes, investigation of how these components differentially interrelate is crucial for optimizing infant wellbeing. Despite research supporting the diathesis‐stress and differential susceptibility hypotheses, no studies have yet addressed potential interactions between temperament and socioemotional outcomes as moderated by mind‐mindedness. Mind‐mindedness was multidimensionally assessed using both appropriate and non‐attuned mind‐related comments, as evidence suggests both dimensions affect socioemotional wellbeing (Colonnesi et al. 2019; Meins et al. 2012; Shai and Meins 2018; Silletti et al. 2022; Zeegers et al. 2017).

The first aim of this study was to examine the influence of maternal mind‐mindedness on infant socioemotional development. Corresponding with previous research linking mind‐minded parenting practices to later socioemotional outcomes (Meins et al. 2013; Colonnesi et al. 2019; Hughes et al. 2017), it was hypothesized that (a) greater appropriate mind‐related comments would be associated with higher socioemotional wellbeing and that (b) greater non‐attuned comments would be associated with lower socioemotional wellbeing.

The second aim of this study was to explore the potential moderating role of maternal mind‐mindedness on the relationship between infant temperament and socioemotional wellbeing. Temperament was examined with separate measures for negative affect, surgency, and effortful control. Consistent with temperament research associating varying developmental outcomes with particular parenting practices (e.g., Zilanawala et al. 2019), it was hypothesized that greater appropriate mind‐related comments and greater non‐attuned comments would each moderate the relationship between socioemotional wellbeing and; (a) negative affect (as suggested by McMahon and Newey 2018; Planalp et al. 2019; Zhang et al. 2022), (b) surgency (akin to Brown et al. 2022; del Puerto‐Golzarri et al. 2022; Ruiz‐Ortiz et al. 2023), and (c) effortful control (resonant with Nikolić et al. 2022; Eisenberg et al. 2015; Feldman and Olson 2021; Lengua et al. 2019; Slagt et al. 2016). Although rare in literature, studies involving greater non‐attuned comments with poorer developmental outcomes also informed these hypotheses (Meins et al. 2018; Nikolić et al. 2022; Planalp et al. 2019).

## 2 Method

### 2.1 Participants

Drawn from a larger sample of a broader study investigating influences upon infant wellbeing, infants were approximately four‐ or 8‐months‐old. One dyad was excluded from the study due to a conflict of interest. Thus, participants comprised 80 mothers and their infants (39 boys, 41 girls). Mothers were aged between 19 and 43 years (*M* = 32.70, SD = 4.64, range = 19.53–43.46). Infants were aged between 98 and 269 days (*M* = 182.01, SD = 62.57) and included two groups: a 4‐month‐old group (*M* = 128.41, SD = 58.61, range = 98–152) and an 8‐month‐old group (*M* = 239.65, SD = 15.14, range = 214–269). Dyads were also culturally and socioeconomically diverse, resided in urban or suburban communities, and were recruited from Sydney, Australia. Most mothers were in married or committed cohabiting relationships (92.50%), suggesting that infants were most likely cared for within two‐parent households. Socioeconomic status was indexed using the Socio‐Economic Indexes for Areas (SEIFA) by the Australian Bureau of Statistics (ABS). Specifically, the Index of Relative Socioeconomic Disadvantage (IRSD) was used, which provides scores ranging from 1 to 10; higher scores indicate a lower level of disadvantage, and lower scores indicate a higher level of disadvantage. IRSD scores ranged between 1 and 10 (*M* = 6.20, SD = 3.15; *n* = 76, *n*_missing_ = 4). Key demographic information, including maternal ethnicity and education, is shown in Table 1. Of the 80 dyads, 63 were included in the final analyses due to exclusions relating to non‐English maternal speech (*n* = 10), technical issues (*n* = 6), and incomplete task participation (*n* = 1).

**TABLE 1:** Demographic characteristics (*n* = 80).

Characteristic	*n*	%
Maternal ethnicity		
White European	40	50.00
Middle‐Eastern	5	6.25
South‐East Asian	11	13.75
South Asian	8	10.00
East Asian	12	15.00
Aboriginal/Torres Strait Islander	1	1.25
Hispanic/Latino	1	1.25
African/African‐American	2	2.50
Maternal education		
Year 12 or equivalent	5	6.25
TAFE qualification or trade apprenticeship	12	15.00
Undergraduate university degree	35	43.75
Postgraduate university degree	28	35.00
Maternal marital status		
Married	63	78.75
Engaged	5	6.25
Separated	5	6.25
Committed relationship (not cohabitating)	1	1.25
De facto relationship (cohabitating)	6	7.50

Abbreviation: TAFE, Technical and further education.

The study was designed and conducted consistent with ethical standards defined in the Declaration of Helsinki. Informed written consent was gained from the mother of each infant before assessment or data collection occurred. Ethical approval was obtained for all procedures involving human participants, granted by the Western Sydney University Human Research Ethics Committee in Sydney, Australia.

### 2.2 Procedure

Ethics approval was gained through the Western Sydney University Ethics Committee. Mothers and their infants were recruited through Western Sydney University's MARCS BabyLab Register. This study was not preregistered. As part of the larger study and prior to attending the laboratory, mothers provided informed consent. Within the broader study, mothers provided informed consent and completed a questionnaire online, which among other measures included demographic information, the Infant Behavior Questionnaire Revised‐Very Short Form (IBQ‐R‐VSF), and the Ages and Stages Questionnaire‐Social‐Emotional (ASQ‐SE). Dyads underwent a block of two eye‐tracking tasks followed by a block of two dyadic‐interaction tasks (still‐face paradigm and free‐play task). However, only the free‐play task was used for the current study.

As per manualised instructions by Meins and Fernyhough (2015), mothers and infants participated in a free‐play session where a range of age‐appropriate toys were provided. While the manual suggests longer sessions typically lasting 20 min, other researchers have opted for shorter sessions lasting 5 min with infants younger than 12 months old (Colonnesi et al. 2019). In the current study, mothers were instructed to play with their babies as they would at home for 4 min. Interactions were recorded through multiple cameras positioned around the room, with infants seated in a bouncer or high chair facing their mothers. Mothers were given a box of toys and instructed that the toys were available to play with but that their use was not mandatory. This coincides with prior research by Laranjo et al. (2010), who assessed mind‐mindedness in free‐play sessions with and without toys. Findings revealed that mind‐related comment frequency was greater with toys when compared to without toys. Thus, in the current study, toys were provided to encourage mothers to engage in more mind‐related communication with their babies.

Following completion, mothers were compensated with a voucher and infants received a toy. Study materials for the mind‐mindedness coding for this study are available from the corresponding author. Data are not available due to ethical requirements.

### 2.3 Measures

#### 2.3.1 Infant Socioemotional Wellbeing

Infant socioemotional wellbeing was measured via the Ages and Stages Questionnaire: Social‐Emotional (ASQ:SE; Squires et al. 2002), using the 22‐item 6 Month Questionnaire (i.e., age‐appropriate for 3‐ to 9‐month‐old infants). Domains assessed cover competency‐based and problem‐based socioemotional behaviors. Specifically, this screening measure assesses parental reports of social and emotional development across seven behavioral areas (i.e., affect, autonomy, adaptive functioning, compliance, social communication, social interaction, and self‐regulation). Mothers reported the degree to which their infant ordinarily demonstrated key behaviors on 19 items using a 3‐point scale (*Most of the time*, *Sometimes*, *Rarely or never*). Scales encompass both positive, competency‐based (e.g., “Does your baby smile at you and other family members?”), and negative, problem‐based (e.g., “Does your baby cry for long periods of time?”) behaviors. An aggregate problem score is generated by summing behavior frequencies of positive items (0 = *Most of the time*, 5 = *Sometimes*, 10 = *Rarely or never*) and negative items (10 = *Most of the time*, 5 = *Sometimes*, 0 = *Rarely or never*), which correspond with age‐dependent cut‐off scores. An additional item response indicates parental concern and contributes to the composite score (5 = *Check if this is a concern*). Total scores encompass competency‐based and problem‐based socioemotional behaviors. Previous research has shown good psychometrics (Squires et al. 2002). In the current study, Items 1 to 19 contributed to the total score; Items 20 to 22 were qualitative in nature and not included in scoring. Cronbach's alpha for scored items produced acceptable internal reliability (*α* = 0.67).

### 2.4 Infant Temperament

Infant temperament was measured using the 37‐item Infant Behavior Questionnaire–Revised Very Short Form (IBQ‐R‐VSF; Putnam et al. 2014). Subscales include negative affect, surgency, and effortful control; greater averages reflect greater factor endorsement. Mothers reported along a seven‐point Likert scale (0 = *Does not apply*, 1 = *Never*, 2 = *Very rarely*, 3 = *Less than half the time*, 4 = *About half the time*, 5 = *More than half the time*, 6 = *Almost always*, 7 = *Always*). Higher ratings reflected stronger endorsement of items within the relevant subscale. Previous research has shown good psychometrics (Putnam et al. 2014) and suitability for ethnically diverse samples (Leerkes et al. 2017). Cronbach's alpha for the current study yielded good internal reliabilities: negative affect (*α* = 0.86), surgency (*α* = 0.86), and effortful control (*α* = 0.73).

### 2.5 Maternal Mind‐Mindedness

Maternal mind‐mindedness was coded from recordings of dyadic interactions during a free‐play task with toys lasting 4 min. Infants were seated in a bouncer or highchair facing their mothers. Speech was transcribed verbatim and subsequently classified whilst observing the videos according to the Mind‐Mindedness Coding Manual (Meins and Fernyhough 2015).

All mother‐spoken comments were coded as mind‐related or not mind‐related. Criteria for mind‐related comments are outlined in Table 2. Meins and Fernyhough (2015) outline that comments are considered mind‐related if they use “an explicit internal state term to comment on what the infant may be thinking, experiencing, or feeling” (p. 5). Comments referencing the inner world of infants must correspond with (a) desires and preferences (i.e., comments contain words such as like, dislike, preference, want, favorite, hate); (b) cognitions (i.e., comments contain words such as decide, know, recognize, remember, interested, notice, focused, fascinated, curious); (c) emotions (i.e., comments contain words such as shy, solemn, happy, sad, scared, serious, grumpy, moody, confused, excited); (d) epistemic states (i.e., comments contain words such as teasing, joking, playing games with me); or (e) talking on the infant's behalf (i.e., comments intended to be spoken or thought by the infant).

**TABLE 2:** Coding criteria for mind‐related comments.

Comment category	Example quote
Desires and preferences	“You like noisy things.” “You want this toy.”
Cognitions	“That doesn't interest you.” “What do you think of that toy?”
Emotions	“You're bored of that toy.” “Are you feeling excited?”
Epistemic states	“You're cheeky.” “Are you teasing me?”
Talking on the infant's behalf	“Everything goes in my mouth.” “What's it doing mummy?”

Coding also considers specificity. For example, “What do you think?” is not coded as mind‐related because it is too ambiguous, whereas, “What do you think of that toy truck?” is coded as mind‐related because it is specific and connects the infant's internal and external experiences.

Coding differentiates comments by a semantic change or second pause and thus can comprise a sentence or a single word. It does not distinguish between statements and questions; both utterances are considered mind‐related if they meet the aforementioned criteria (see Meins 2013). For example, in response to a child pointing to a book, a comment such as “Do you want to read the book?” is regarded as an appropriate mind‐related comment because it accurately interprets the internal mental state with the external behavior of the child.

Mind‐related comments were subsequently deemed *appropriate* (i.e., accurate; see Table 3) or *non‐attuned* (i.e., inaccurate; see Table 4) in accordance with observed behaviors of mothers and infants on the recordings. Comments were tallied as frequency scores for analyses (i.e., total appropriate and non‐attuned comments). This approach aligns with McMahon and Bernier (2017). This review suggests frequency scores are a valid measure of mind‐mindedness as infants are exposed to mind‐minded comments regardless of any additional comments made, highlighting mixed results between the usage of frequency versus proportional scores (Meins et al. 2003, 2013) and alignment between frequency counts and theoretical outcomes of mind‐mindedness (Bernier et al. 2016; Demers et al. 2010; Laranjo et al. 2010).

**TABLE 3:** Coding criteria for appropriate comments.

Criteria	Example	
	Maternal comment	Infant action
Accurately interpreted internal infant states	“Do you want to feel this toy?”	Infant stretches out hands to touch toy
	“You're such a happy girl!”	While infant is smiling or laughing
	“The rattle is your favorite, isn't it?”	Following infant exhibiting repeated preference for playing with the rattle
Connected current infant activities and states with similar past or future events	“Do you remember we saw a monkey at the zoo?”	While infant is playing with a toy monkey
Guided the infant with how to proceed following interactional lulls	“Do you want to play with this toy?”	Following infant not being focused on any specific items or events

**TABLE 4:** Coding criteria for non‐attuned comments.

Criteria	Examples	
	Maternal comment	Infant action
Inaccurately interpreted internal infant states	“You're so grumpy.”	Infant is exhibiting positive affect
References past or future events unrelated to current infant activities or states	“Do you want to visit your cousin tomorrow?”	Current play or discussion irrelevant to cousin, not previously mentioned
Asked what the infant wanted to do or suggested a new activity despite the infant showing engagement with their current activity	“Would you like to play with this toy instead?”	Infant is enjoying playing with the current toy
Projected internal states relevant to the mothers that were not implied by infant behaviors	“Are you thinking about your sister who you love so much?”	Infant behavior does not imply internal state
Contained ambiguous referents	“You don't like that.”	Infant is not engaged with any specific item or event

To establish interrater reliability, a secondary coder, blinded to the hypotheses, was trained by the primary coder using example recordings of final sample infant‐mother dyads. During this training phase, disagreements between coders were resolved through discussion while reviewing the recordings. Following this training phase, the secondary coder independently coded randomly selected dyadic interactions within the free‐play phase (*n* = 17, 27%). Interrater reliability was examined using intraclass correlations (ICC). Excellent agreement was demonstrated between the primary and secondary coders for this data subset; ICC average measure for appropriate comments was 0.992 with a 95% confidence interval from 0.978 to 0.997 (*F* [16, 16] = 123.684, *p* < 0.001) and for non‐attuned comments was 0.951 with a 95% confidence interval from 0.865 to 0.982 (*F* [16, 16] = 20.467, *p* < 0.001).

### 2.6 Analytical Plan

This study employed a cross‐sectional quantitative design. Selective attrition was analyzed using independent *t*‐test and chi‐squared analyses. Descriptive statistics and assumption checks were executed, with necessary transformations applied.

To address the first aim, assessing whether maternal mind‐mindedness was associated with better socioemotional outcomes, bivariate correlations were performed between mind‐related comment variables and wellbeing.

To address the second aim, evaluating possible moderating influences of maternal mind‐mindedness on the relationship between infant temperament and wellbeing, two hierarchical regression analyses were conducted. As the selected infancy stages are characterized by attentional control (Lewis and Carpendale 2014; Mundy 2013; Rothbart et al. 2011) and intentional communication developments (Colonnesi et al. 2019), infant age was controlled in both regression models. Given possible correlations with mind‐mindedness (McMahon and Bernier 2017), sensitivity (Akkus et al. 2020; Alvarenga et al. 2020; Bornstein et al. 2020), and verbosity (Meins et al. 2002), maternal education was also controlled in both regression models. Further, as maternal education was significantly correlated with the total number of comments in the current study, it acted as a proxy variable to account for verbosity.

Control variables were entered in the first step (infant age and maternal education). Temperament subscales were entered in the second step (negative affect, surgency, effortful control). Mind‐mindedness was included in the third step (appropriate comments or non‐attuned comments). Finally, interaction terms between mind‐mindedness and each temperament subscale were added in the fourth step. Post‐hoc supplementary investigations were also performed to elucidate the characteristics of significant moderation effects using simple slopes analyses. An alpha of 0.05 was used for all analyses.

### 2.7 Statistical Power

Adequate statistical power for identifying any existing effects was conferred and considered from multiple aspects. Stevens (2002) specifies a ratio of 15:1 (participants to predictor variables). Given the use of four predictor variables in the current study, the desired sample size was estimated at 60 dyads. G*Power software computing a priori power analyses supported this estimation, indicating that for small‐medium effect sizes, a minimum of 56 parent‐infant dyads was required (> 0.20). Calculations also coincided with similar power approximations by Colonnesi et al. (2019). Further evidence for sample size was found in recent infant mind‐mindedness studies, which have shown significant main and interaction effects with sample sizes ranging from 44 dyads (Giovanelli et al. 2020) to 93 dyads (Silletti et al. 2022).

## 3 Results

### 3.1 Attrition Analyses

Attrition bias was examined between those included (*n* = 63) and excluded from (*n* = 17) the final sample. Reasons for exclusion entailed (a) during the free‐play task, maternal speech was predominantly in a language other than English limiting the possibility of engaging in mind‐mindedness coding in English, *n* = 10; (b) technical issues with recordings or saving recordings, *n* = 6; or (c) insufficient task completion, *n* = 1. A significant difference was found for maternal ethnicity (*X* ^2^ [1, *N* = 80] = 9.04, *p* = 0.003), with fewer diverse dyads included in the final sample compared to White European dyads. A significant difference for primary language spoken at home (*X* ^2^ [1, *N* = 80] = 31.40, *p* < 0.001) was also found. The proportion of non‐English‐speaking households in the final sample was significantly less compared to English‐speaking households. No significant differences were found for infant age, infant sex, infant ethnicity, maternal education, maternal employment, maternal marital status, and maternal age (*p*s > 0.05).

### 3.2 Preliminary Analyses

Descriptive statistics are depicted in Table 5.

**TABLE 5:** Descriptive statistics (*n* = 63).

Variable	*M*	SD	Range
Negative affect (IBQ‐R‐VSF)	46.24	13.61	19–76
Surgency (IBQ‐R‐VSF)	52.13	16.46	18–83
Effortful control (IBQ‐R‐VSF)	62.21	9.11	40–76
Appropriate mind‐minded comments	5.14	3.63	0–15
Non‐attuned mind‐minded comments	1.73	2.76	0–15
Socioemotional wellbeing (ASQ: SE)	25.63	19.62	0–85

Normality checks revealed that socioemotional wellbeing was positively skewed (histogram; Shapiro‐Wilk, *t* [63] = 3.65 *p* < 0.05). Thus, socioemotional wellbeing was square root transformed. Both mind‐related comment variables displayed positive skews (histograms; Shapiro‐Wilk_appropriate_, *t* [63] = 2.09, *p* < 0.05; Shapiro‐Wilk_non‐attuned_, *t* [63] = 9.88, *p* < 0.05), with non‐attuned comments also showing high kurtosis. However, as it is unknown whether mind‐mindedness approximates normal distributions within the general population, transformations were not applied. Negative affect and surgency were normally distributed upon histogram inspection. Effortful control was negatively skewed (Shapiro‐Wilk, *t* [63] = 2.13 *p* < 0.05). Effortful control was subsequently transformed via square root and reflection functions.

#### 3.2.1 Correlations

Bivariate correlations are presented in Table 6. Results showed lower socioemotional wellbeing was significantly associated with older infant age, higher maternal education, and lower effortful control. Higher surgency was significantly associated with older infant age, lower effortful control, and higher negative affect. Higher negative affect was also significantly associated with older infant age. Appropriate mind‐related comments were positively correlated with non‐attuned mind‐related comments.

**TABLE 6:** Bivariate correlations between control, independent, and dependent variables.

Variable	Infant age	Maternal education	Negative affect	Surgency	Effortful control	Appropriate comments	Non‐attuned comments	Socioemotional wellbeing
Infant age	—							
Maternal education	0.07	—						
Negative affect	0.37**	−0.15	—					
Surgency	0.63***	−0.12	0.48***	—				
Effortful control	−0.03	0.10	−0.17	−0.40***	—			
Appropriate comments	−0.18	0.13	−0.21	−0.20	0.08	—		
Non‐attuned comments	−0.21	0.13	−0.04	−0.21	0.09	0.33**	—	
Socioemotional wellbeing	−0.31*	−0.26*	−0.02	−0.24	0.30*	−0.11	0.10	—

*Note:* Negative affect, Surgency and effortful control were captured via the IBQ‐R‐VSF, Socioemotional wellbeing was captured via the ASQ: SE. *p < 0.05, **p < 0.01, ***p < 0.001.

## 4 Main Analyses

Maternal education was used as a control for verbosity in both models (see Meins et al. 2002).

### 4.1 Mind‐Related Comments and Socioemotional Wellbeing

Socioemotional wellbeing was not significantly related to appropriate comments.

(*r* = −0.109, *p* = 0.397) nor non‐attuned comments (*r* = 0.100, *p* = 0.434).

### 4.2 Mind‐Related Comments and Temperament on Socioemotional Wellbeing

#### 4.2.1 Appropriate Mind‐Related Comments and Temperament on Socioemotional Wellbeing

**FIGURE 1:**
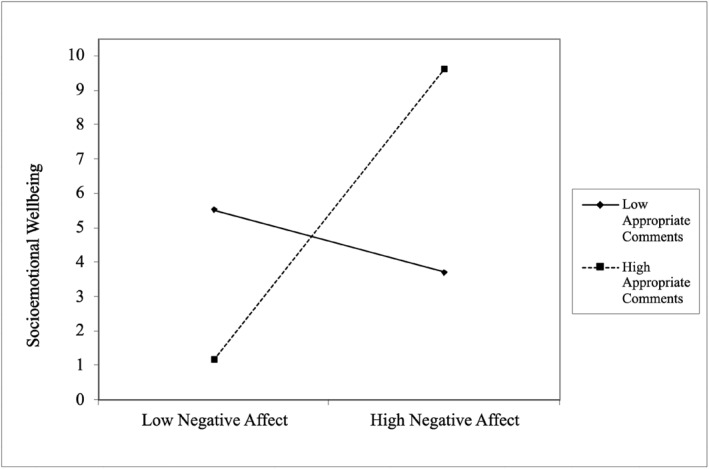
Simple slopes between infant negative affect and appropriate mind‐related comments on infant socioemotional wellbeing (*n* = 63).

As shown in Table 7 (in Step 4), greater socioemotional wellbeing was predicted by younger infant age, greater effortful control, and the interaction between negative affect and appropriate comments, which explained 34% variance (*R* ^2^ = 0.34, adjusted *R* ^2^ = 0.23, *F* (9, 53) = 3.01, *p* = 0.006). Older infant age significantly predicted poorer socioemotional wellbeing (*β* = −0.35, *p* = 0.031). For each one‐point change in effortful control, socioemotional wellbeing increased by 0.62 (*β* = 0.38, *p* = 0.007). As shown in Figure 1 (and Table 7), the interaction between negative affect and appropriate comments also significantly predicted socioemotional wellbeing (*β* = 0.32, *p* = 0.032). Infants with higher negative affect whose mothers provided higher appropriate comments had significantly better socioemotional wellbeing than infants with lower negative affect whose mothers provided higher appropriate comments (simple slopes = 0.148, *t* = 2.31; *p* = 0.024). There was no significant difference between infants with higher negative affect and lower negative affect when mothers provided lower appropriate comments (simple slopes = −0.032, *t* = 1.39, *p* = 0.170). No other interactions were significant.

**TABLE 7:** Hierarchical multiple regression predicting socioemotional wellbeing from temperament and appropriate comments (*n* = 63).

Step	*B* (SE *B*)	*β*	*t*	*R* ^2^ (Adjusted *R* ^2^)	∆*R* ^2^
Step 1				0.15 (0.12)**	0.15**
Infant age	−1.16 (0.48)	−0.29	2.41*		
Maternal education	−0.55 (0.28)	−0.24	1.97		
Step 2				0.26 (0.19)**	0.11*
Infant age	−1.35 (0.64)	−0.34	2.11*		
Maternal education	−0.58 (0.28)	−0.25	2.08*		
Negative affect	0.02 (0.02)	0.12	0.89		
Surgency	0.00 (0.02)	0.02	0.12		
Effortful control	0.55 (0.21)	0.34	2.59*		
Step 3				0.28 (0.20)**	0.02
Infant age	−1.42 (0.64)	−0.35	2.22*		
Maternal education	−0.54 (0.28)	−0.23	1.93		
Negative affect	0.02 (0.02)	0.10	0.75		
Surgency	0.00 (0.02)	0.02	0.09		
Effortful control	0.56 (0.21)	0.34	2.63*		
Appropriate comments	−0.08 (0.07)	−0.15	1.23		
Step 4				0.34 (0.23)**	0.06
Infant age	−1.40 (0.63)	−0.35	2.21*		
Maternal education	−0.45 (0.28)	−0.19	1.63		
Negative affect	0.03 (0.02)	0.19	1.40		
Surgency	0.00 (0.02)	−0.02	0.93		
Effortful control	0.62 (0.22)	0.38	0.01**		
Appropriate comments	0.01 (0.58)	0.01	0.01		
Negative affect × appropriate comments	0.01 (0.01)	0.32	2.21*		
Surgency × appropriate comments	−0.01 (0.01)	−0.14	0.96		
Effortful control × appropriate comments	0.00 (0.01)	−0.12	0.11		

*Note:* Negative affect, Surgency and effortful control capture via the IBQ‐R‐VSF, Socioemotional wellbeing captured via the ASQ: SE. *p < 0.05, **p < 0.01, ***p < 0.001.

#### 4.2.2 Non‐Attuned Mind‐Related Comments and Temperament on Socioemotional Wellbeing

Mahalanobis distance scores indicated that one multivariate outlier was unacceptable (*X* ^2^ = 27.50, df = 6, *p* = 0.001, *n* = 62). It was therefore removed to prevent biasing data across multiple variables.

As shown in Table 8 (in Step 4), greater socioemotional wellbeing was predicted by younger infant age and greater effortful control, which explained 30% variance (*R* ^2^ = 0.30, adjusted *R* ^2^ = 0.18, *F* (9, 52) = 2.48, *p* = 0.019). Older infant age significantly predicted poorer socioemotional wellbeing (*β* = −0.39, *p* = 0.033). For each one‐point change in effortful control, socioemotional wellbeing increased by 0.63 (*β* = 0.39, *p* = 0.011). No significant effects were found for maternal education, negative affect, surgency, or non‐attuned comments. No significant interactions were found between temperamental variables and non‐attuned comments on socioemotional wellbeing.

**TABLE 8:** Hierarchical multiple regression predicting socioemotional wellbeing from temperament and non‐attuned comments (*n* = 62).

Step	*B* (SE *B*)	β	*t*	*R* ^2^ (Adjusted *R* ^2^)	∆*R* ^2^
Step 1				0.15 (0.12)**	0.15**
Infant age	−1.19 (0.49)	−0.29	2.44*		
Maternal education	−0.58 (0.29)	−0.24	2.01*		
Step 2				0.26 (0.19)**	0.11*
Infant age	−1.37 (0.65)	−0.34	2.13*		
Maternal education	−0.59 (0.28)	−0.25	2.09*		
Negative affect	0.02 (0.02)	0.12	0.86		
Surgency	0.00 (0.02)	0.02	0.14		
Effortful control	0.55 (0.21)	0.34	2.55*		
Step 3				0.26 (0.18)**	0.00
Infant age	−1.34 (0.68)	−0.33	1.99		
Maternal education	−0.60 (0.29)	−0.25	2.08*		
Negative affect	0.02 (0.02)	0.11	0.84		
Surgency	0.00 (0.02)	0.03	0.15		
Effortful control	0.55 (0.22)	0.34	2.52*		
Appropriate comments	0.02 (0.12)	0.02	0.18		
Step 4				0.30 (0.18)*	0.04
Infant age	−1.56 (0.72)	−0.39	2.19*		
Maternal education	−0.58 (0.29)	−0.24	1.98		
Negative affect	0.03 (0.02)	0.19	1.31		
Surgency	0.00 (0.02)	0.03	0.17		
Effortful control	0.63 (0.23)	0.39	2.63*		
Non‐attuned comments	−0.04 (0.18)	−0.04	0.23		
Negative affect × non‐attuned comments	0.02 (0.01)	0.22	1.32		
Surgency × non‐attuned comments	−0.01 (0.01)	−0.25	1.15		
Effortful control × non‐attuned comments	0.00 (0.01)	−0.03	0.19		

*Note:* Negative affect, Surgency and effortful control capture via the IBQ‐R‐VSF, Socioemotional wellbeing captured via the ASQ: SE. *p < 0.05, **p < 0.01, ***p < 0.001.

## 5 Discussion

This study aimed to investigate the relationship between maternal mind‐mindedness, infant temperament, and infant socioemotional wellbeing. Contrary to hypotheses, results indicated that both appropriate and non‐attuned mind‐minded comments were not significantly associated with, or predicted, infant socioemotional wellbeing. Aligning with hypotheses, there was a significant interaction between appropriate mind‐minded comments and negative affect which predicted infant socioemotional wellbeing. Particularly, infants with higher negative affect who received greater appropriate comments demonstrated significantly better socioemotional outcomes when compared to infants with lower negative affect.

Given the profound impact of parental sensitivity on infant development, this study examined how maternal mind‐mindedness and infant temperament shape socioemotional outcomes, both individually and combined. The first aim of this study was to explore the relationship between maternal mind‐mindedness and infant socioemotional wellbeing. Contradicting hypotheses, neither appropriate nor non‐attuned mind‐related comments were associated with wellbeing. Additionally, neither mind‐mindedness variable independently predicted wellbeing. Results contrast previous research endorsing mind‐mindedness as influencing advantageous outcomes (Aldrich et al. 2021; McMahon and Bernier 2017; Zeegers et al. 2017). Results also contrast recent longitudinal studies where associations between increased appropriate mind‐mindedness during infancy and decreased socioemotional problems during toddlerhood were found (Hobby et al. 2022; Laflamme et al. 2022). Explanations for null results in the current study are possibly attributable to when measures were administered during development. Developmental consequences of mind‐mindedness may manifest with maturation, therefore becoming more measurable. Future studies may benefit from extended wellbeing monitoring to clarify whether relationships between mind‐mindedness during infancy and socioemotional outcomes during childhood fluctuate over time.

The second aim of this study was to explore the moderating role of maternal mind‐mindedness on the relationship between infant temperament and socioemotional wellbeing. Consistent with hypotheses, appropriate mind‐related comments were found to interact with negative affect to predict wellbeing. Specifically, infants with higher negative affect who received greater appropriate comments had significantly better socioemotional wellbeing than infants with lower negative affect who received greater appropriate comments. When given more appropriate comments, higher negative affect infants demonstrated enhanced outcomes compared to lower negative affect infants. Conversely, no significant socioemotional differences were observed between higher and lower negative affect infants who received fewer appropriate comments.

Corroborating differential susceptibility hypothesis research, these findings align with previous studies investigating differential relationships between temperament and wellbeing (Belsky and Pluess 2009). Notably, Zilanawala et al. (2019) found that infants with predispositions toward internalizing or externalizing problems experienced different trajectories depending upon parental appropriate comment usage. Similarly, Zeegers et al. (2017) found that appropriate commentary differentially impacted infants according to psychopathological susceptibilities. Relatedly, Davies et al. (2021) found that dove temperaments, characterized by greater sensitivity to environments, can more easily adapt behavior depending upon the situation. Similarly, findings in the current study may suggest that higher negative affect infants have heightened susceptibility to the protective or detrimental effects of nurturing versus aversive social environments.

Current results also complement findings that infants with higher negative affect surpass those with lower negative affect in self‐regulation measures within securely attached dyads (Kim and Kochanska 2012) and that children with elevated negative emotionality are favorably impacted by positive parenting (Slagt et al. 2016). Higher negative affect temperaments may possess attributes that heighten receptivity to sensitive parenting, whereby appropriate comments potentially function as an important strategy.

Inconsistent with hypotheses, interactions were not found between non‐attuned comments and negative affect. This was unexpected, given previous research highlighting that insensitive parenting during infancy compounds later behavioral dysregulation in childhood for higher negative affect individuals (Zhang et al. 2022). Non‐attuned comments are sporadically reported in mind‐mindedness research (McMahon and Bernier 2017); small but insignificant associations have been reported between non‐attuned comments and sensitivity (Licata et al. 2014; Meins et al. 2003, 2012). Given the lower prevalence of non‐attuned comments in the current sample as compared to appropriate comments, it is plausible that non‐attuned comments are too infrequent to significantly influence infant socioemotional wellbeing. Alternatively, perhaps dyadic exchanges following rupture (i.e., non‐attuned) and repair (i.e., appropriate) cycles rectify the detrimental consequences of non‐attuned comments (Meins 2013). It is possible that incorporating clinical populations in future research may allow for the examination of these possibilities. This is because clinical populations may exhibit a greater frequency of non‐attuned comments, amplified due to possible challenges impacting mind‐minded parenting (e.g., financial stress, mental health diagnoses, poor attachment relationships).

Contrary to hypotheses, interactions were null between surgency and appropriate or non‐attuned comments. Such results were unexpected because higher surgency and negative parenting have been associated with externalizing issues in toddlers (Brown et al. 2022) and aggression in children (Ruiz‐Ortiz et al. 2023), while higher surgency and positive parenting have been associated with optimized childhood executive functioning (Suor et al. 2019). Surgency did not independently predict wellbeing, conflicting with research regarding optimal levels implicated with ideal childhood outcomes (Borowski et al. 2021; Laible et al. 2014). Conceivably, surgency may not exert influence during early development. Further research during toddlerhood and childhood may reveal clearer relationships once temperament‐disorder trajectories are underway.

Diverging from hypotheses, interactions were also null between effortful control and appropriate or non‐attuned comments. These results were unexpected as parental sensitivity has been shown to enhance high effortful control advantages (Neppl et al. 2020), whereas parental insensitivity has been shown to exacerbate low effortful control disadvantages (Slagt et al. 2016). Current results showed that lower effortful control independently predicted poorer socioemotional wellbeing within both regression models. This aligns with previous research linking effortful control with adverse childhood outcomes (Kostyrka‐Allchorne et al. 2020; Yavuz‐Müren et al. 2022Yavuz‐Müren et al. 2022). It may be that this temperament trait robustly predicts wellbeing such that any contributions of mind‐mindedness are rendered negligible. Subsequent studies should examine whether other moderators may influence the relationship between effortful control and wellbeing; such findings could inform interventions.

In this study, it was also evident that there was a positive correlation between appropriate and non‐attuned mind‐related comments. This was unexpected and is contrary to the findings in most mind‐mindedness studies, contrasting with previous research (Meins et al. 2012; Colonnesi et al. 2019). A possible explanation for this divergence from existing literature is that the paradigm in the present study used to facilitate dyadic interaction was contextually stressful; unfamiliar laboratory setting, presence of recording equipment, residual arousal from previous tasks not analyzed in this study. These factors may have promoted disruption to typical social exchanges and influenced the correlation between appropriate and non‐attuned comments. McMahon and Newey (2018) have similarly speculated that non‐attuned comment frequency may increase in stressful situations because of attachment system activation for both mother and infant. Observation of dyads in natural settings may be considered for future research. Alternatively, this finding may reflect mothers who are more expressive in dyadic interactions tending to increase their use of mind‐minded comments overall, both accurately (i.e., appropriate comments) and inaccurately (i.e., non‐attuned comments) when attempting to discern the internal states of their infants. Further research to explore this intersection between verbosity and comment type is recommended.

Beyond the hypothesized relationships, control variable effects emerged. Younger infant age independently predicted better wellbeing throughout each regression. Younger infants typically have greater care requirements than older infants, therefore it is plausible that participation was influenced by infant wellbeing and practical considerations involving infant care (e.g., timing of naps/feeds, ease of travel). Additionally, higher maternal education independently predicted poorer infant wellbeing within each regression. It is feasible that mothers who are motivated to participate in infant research are more educated and have more concerns about their infants' wellbeing.

While the current study did not specifically focus on the interplay between mind‐mindedness and cultural diversity, it may provide insight into this relationship. Previous research investigating this relationship is severely limited. Hughes et al. (2017) explored this aspect; Hong Kong parents demonstrated lower mind‐mindedness compared to UK parents, which was linked to differences in theory of mind measures. Wang et al. (2017) suggested an underlying mechanism for this relationship; cultural values of social conformity rather than individual autonomy may influence the manifestation of mind‐minded parenting and, consequently, also socioemotional outcomes. Further research should further explore these important perspectives.

Although this study provides a novel contribution to research, specifically that higher negative affect infants demonstrated better wellbeing when exposed to greater appropriate comments compared to lower negative affect infants, there are some limitations. Firstly, the data were collected cross‐sectionally. Whilst temperament traits are thought to manifest early in infancy and remain stable over time, it is conceivable that mothers' views of their infants' temperament were influenced by how they currently perceived their infants' socioemotional wellbeing, or that mothers' views of how they perceive their infants' socioemotional wellbeing is influenced by how they perceive their infants' temperament. Furthermore, the temporal order of effects cannot be determined. Future research should instead consider longitudinal data. Secondly, while the study maintained diverse cultures, attrition slightly reduced this diversity within the final sample. If attrition had not occurred, cultural diversity would have been stronger; prospective studies could address this obstacle by requesting that during the task mothers speak to their infants in English. Thirdly, despite using validated wellbeing questionnaires, supplementing parental‐report questionnaires with observational data may improve reliability.

Despite these limitations, this study offers several strengths. It pioneers the exploration of interactions between extrinsic mind‐mindedness influences and intrinsic temperament factors because such investigations are absent from research. Hence, it also illuminates nomothetic and idiographic comparisons commonly lacking in other studies, that is, general commonalities and individual differences among participants (Pérez‐Edgar et al. 2020). Findings also extend extant mind‐mindedness literature, implicating both theoretical and practical advances. Behavioral coding with excellent interrater reliability was used to capture mind‐mindedness variables; agreement in the current study is an improvement upon previous mind‐mindedness literature (Giovanelli et al. 2020; Hobby et al. 2022; Silletti et al. 2022). Moreover, mind‐mindedness was considered multidimensionally, which provided the opportunity to isolate comment typology effects. Controlling for infant age and maternal education prevented confounding factors and enhanced model accuracy. Lastly, despite the slight reduction in cultural diversity due to attrition, the study maintained cultural diversity compared to other recent studies (Meins et al. 2019; Planalp et al. 2022).

This study holds both theoretical and practical implications. Theoretically, findings support the differential susceptibility hypothesis whereby certain dispositions are especially responsive to environmental stimuli, including parenting (Belsky and Pluess 2009). Infants with higher negative affect benefited disproportionately from higher appropriate comment frequency but did not suffer disproportionately from lower appropriate comment frequency. This implies hope for families of infants with higher negative affect, but also suggests appropriateness for further research investigating later developmental stages using age‐appropriate instruments. Practically, findings may promote strategies for protecting and enhancing wellbeing because negative affect generates genetic vulnerability to internalizing and externalizing disorders (Sirois et al. 2019), and, also transdiagnostically increases later psychopathology risk (Kostyrka‐Allchorne et al. 2020). Using appropriate mind‐related comments may empower parents with actionable strategies to pacify difficulties accompanying negative emotionality, such as mind‐mindedness early parenting programs. This may resultantly facilitate improvements in socioemotional outcomes for infants.

## 6 Conclusion

Overall, this study highlights that mind‐minded parenting may impact interactions between infant temperament and socioemotional wellbeing. Specifically, when exposed to greater appropriate comments, infants with higher negative affect socioemotionally thrived compared to infants with lower negative affect. Current findings lend support for the differential susceptibility hypothesis, with mind‐mindedness emerging as an adaptive function shaping developmental outcomes for vulnerable infants who have higher negative affect. Additionally, effortful control predicted wellbeing suggesting that certain temperaments experience optimal socioemotional development regardless of mind‐mindedness. Conversely, surgency did not seemingly influence wellbeing in infancy. Importantly, findings facilitate theoretical and practical implications that may meaningfully shift influential forces during critical developmental stages. Future research using longitudinal observations and mixed measures will deepen understanding concerning how mechanisms underpinning temperament and parenting reciprocally shape wellbeing.

## Author Contributions

**Allira Doyle:** conceptualization, data curation, formal analysis, methodology, visualization, writing – original draft, writing – review and editing. **Emma E. Walter:** conceptualization, supervision, writing – review and editing. **Samudra Radhakrishnan:** data curation, investigation, methodology, project administration, validation. **Frances L. Doyle**: conceptualization, data curation, funding acquisition, investigation, methodology, project administration, resources, supervision, visualization, writing – review and editing.

## Conflicts of Interest

The authors declare no conflicts of interest.

## Data Availability

Study materials for the mind‐mindedness coding for this study are available from the first or corresponding author. Data are not available due to ethical requirements.
